# Parietal memory network and memory encoding versus retrieval impairments in PD‐MCI patients: A hippocampal volume and cortical thickness study

**DOI:** 10.1111/cns.70062

**Published:** 2024-10-08

**Authors:** Serhat Sahin, Halil Aziz Velioglu, Burak Yulug, Zubeyir Bayraktaroglu, Suleyman Yildirim, Lutfu Hanoglu

**Affiliations:** ^1^ Functional Imaging and Cognitive‐Affective Neuroscience Lab (fINCAN) Health Sciences and Technology Research Institute (SABITA), Istanbul Medipol University Istanbul Turkey; ^2^ Center for Psychiatric Neuroscience Feinstein Institute for Medical Research Manhasset New York USA; ^3^ Department of Neurology and Clinical Neuroscience, School of Medicine Alanya Alaaddin Keykubat University Alanya Turkey; ^4^ Department of Microbiology, School of Medicine Istanbul Medipol University Istanbul Turkey; ^5^ Department of Neurology, School of Medicine Istanbul Medipol University Istanbul Turkey

**Keywords:** cortical thickness, encoding failure, gray matter volume, hippocampus, parietal memory network, Parkinson's disease‐mild cognitive impairment, retrieval failure

## Abstract

**Objective:**

The pathophysiology behind memory impairment in Parkinson's Disease Mild Cognitive Impairment (PD‐MCI) is unclear. This study aims to investigate the hippocampal and cortical atrophy patterns in PD‐MCI patients with different types of memory impairments, categorized as Retrieval Failure (RF) and Encoding Failure (EF).

**Methods:**

The study included 16 healthy controls (HC) and 34 PD‐MCI patients, divided into RF (*N* = 18) and EF (*N* = 16) groups based on their Verbal Memory Processes Test (VMPT) scores, including spontaneous recall, recognition, and Index of Sensitivity to Cueing (ISC). Hippocampal subfields and cortical thicknesses were measured using the FreeSurfer software for automatic segmentation.

**Results:**

Compared to the HC group, the EF group exhibited significant atrophy in the left lateral occipital region and the right caudal middle frontal, superior temporal, and inferior temporal regions (p⟨0.05). The RF group displayed significant atrophy in the left lateral occipital, middle temporal, and precentral regions, as well as the right pars orbitalis and superior frontal regions (p⟨0.05). Hippocampal subfield analysis revealed distinct volume differences between HC‐EF and RF‐EF groups, with significant reductions in the CA1, CA3, and CA4 subregions in the EF group, but no differences between HC and RF groups (*p* > 0.05).

**Conclusion:**

Gray matter atrophy patterns differ in PD‐MCI patients with encoding and retrieval memory impairments. The significant hippocampal atrophy in the EF group, particularly in the CA subregions, highlights its potential role in disease progression and memory decline. Additionally, the convergence of atrophy in the lateral occipital cortex across both RF and EF groups suggests the involvement of the Parietal Memory Network (PMN) in PD‐related memory impairment.

## Graphical abstract

This study explores the distinct patterns of hippocampal and cortical atrophy in Parkinson's Disease Mild Cognitive Impairment patients, categorized by Retrieval Failure and Encoding Failure memory impairments. Using FreeSurfer software, significant atrophy differences were identified in specific brain regions. The results hint at the potential role of the “Parietal Memory Network” in mediating PD‐related memory issues.

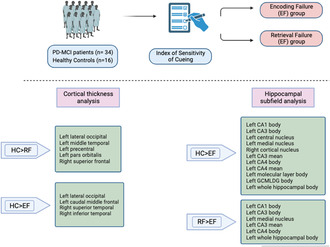

## 1 INTRODUCTION

Cognitive impairments are commonly observed in patients Parkinson's Disease (PD) without dementia, in addition to other motor symptoms. The presence of cognitive impairments is a significant risk factor for the PD's progression to dementia.^1^ Mild Cognitive Impairment (MCI), which is considered the boundary between dementia and normal aging and does not harm daily life functionality, is common in PD patients.^2^, ^3^ Thirty percent of patients diagnosed with PD meet the diagnostic criteria for MCI.^4^ However, the progression and nature of cognitive impairments in PD are heterogeneous.^1^ Therefore, classifying patients based on the different types of cognitive impairments is crucial to determine the progression to Parkinson's Disease Dementia (PDD).^4^

To explain the cause and type of memory impairments in PD and PD‐MCI, the dual syndrome hypothesis has been proposed. One aspect of this hypothesis is the classical approach seen in PD, which is the impairment of frontal executive functions due to the disruption of dopaminergic activity in the frontostriatal cycle.^5^ This disruption in the cycle hampers the retrieval of learned information rather than the learning and recording of information. This type of memory disorder is hypothesized to be a supplementary deficit to executive function skills.^6^ It has been suggested that these disruptions in frontostriatal executive function skills in PD have limited association with the risk of dementia development.^7^ The other aspect of the hypothesis, which is thought to be more associated with dementia development in PD, is a PD pattern that is characterized by memory, visual–spatial skills, hallucinations, psychiatric symptoms and responds more to cholinergic treatment.^6^, ^7^, ^8^ This cognitive involvement pattern, especially difficulty in recording newly learned information and being insensitive to cues in recognition performance, manifests clinically. Involvement due to cholinergic deficiency of the posterior parietal and superior temporal areas, thought to impede encoding and recording skills from episodic memory processes, appears to be a distinctive factor in the transformation to PD dementia.^6^

Neuroimaging studies have often focused on the medial temporal and frontal lobes; however, there are studies showing correlations between the parietal cortex and memory functions.^9^, ^10^ Meta‐analyses related to encoding and retrieval have associated regions such as the precuneus (PCU), middle cingulate cortex (MCC), and the posterior inferior parietal lobule/dorsal angular gyrus (pIPL/dAG) with memory functions. Research in memory literature indicates that these three regions are involved in memory encoding and retrieval and form a functional network.^11^, ^12^, ^13^, ^14^, ^15^ This network has been named the “parietal memory network” (PMN) by Gilmore et al.^16^ (34).

In conclusion, the memory disorders of PD‐MCI patients, their pathophysiology, and their potential relationships with posterior cortical, hippocampal, and frontal structures have not been sufficiently clarified. The aim of our study is to divide PD‐MCI patients into two groups as suggested by Costa et al.^17^—a group experiencing retrieval dysfunction and a group experiencing encoding dysfunction, based on verbal memory spontaneous (free) recall and recognition scores. We intend to compare the hippocampal sub‐region volumes and cortical thickness measurements of PD‐MCI patients who demonstrate different types of memory disorders with the data of a healthy control group. The objective is to investigate whether there is a difference in the hippocampal and cortical atrophy patterns of these two different PD‐MCI patient groups in terms of these two memory involvements.

## 2 SUBJECTS AND METHODS

### 2.1 Subjects

The study was conducted retrospectively using Magnetic Resonance Imaging (MRI) records and neuropsychological evaluation reports obtained from participants included in previous studies and healthy controls who applied to the Neurology Department Polyclinic of Istanbul Medipol University Faculty of Medicine and were informed with a PD‐MCI diagnosis and their written consents were taken. The study included 34 patients aged between 46 and 81 (18 males, 16 females) and 16 healthy control groups aged between 52 and 79. The study received ethical committee approval with the decision numbered 10840098‐604.01.01‐E.1424 dated 14/01/2020 by the “Istanbul Medipol University Non‐Invasive Clinical Research Ethical Board”.

The study included healthy control individuals and those diagnosed with PD, all of whom were aged over 45. These participants had no history of neurological or psychiatric disorders, nor had they experienced head trauma. Patients included for the PD diagnosis were assessed by a neurology specialist with expertise in movement disorders and the diagnosis of PD was defined according to the criteria of Movement Disorder Society.^18^ The healthy control group comprised individuals with a Mini‐Mental State Examination (MMSE) score of 24 or higher. Patients meeting the PD diagnostic criteria but exhibiting symptoms of dementia along with both healthy and patient groups presenting any additional cause that could impede cognitive evaluation (such as irreparable hearing or visual impairment, etc.) were excluded from the study. Moreover, individuals diagnosed with alcohol or substance addiction or any other neurological or psychiatric disease were also excluded from the study (Table 1).

**TABLE 1:** Comparison of the demographic and clinical characteristics of the groups.

	Healthy Control (16)	Retrieval Failure (18)	Encoding Failure (16)	*p*
	*M + SD*	*M + SD*	*M + SD*	
Age	64.8 ± 7.2	66.9 ± 9.8	69.7 ± 8.7	0.13^d^
Education	6.9 ± 3.0	4.8 ± 4.5	5.2 ± 2.8	0.16^d^
Gender	♀ 9 ♂ 7	♀ 9 ♂ 9	♀7 ♂ 9	0.78^b^
ISC	0.86 ± 0.27	0.96 ± 0.06	0.58 ± 0.16	0.00^d^
MMSE	27 ± 1.5	24.5 ± 2.7	23.8 ± 2.2	0.00^a^
GDS	7.4 ± 7.3	10.7 ± 6.9	9.5 ± 4.4	0.22^d^
MDS‐UPDRS		16.9 ± 8.1	14.6 ± 6.3	0.8^c^
Duration of Disease		6.4 ± 3.9	5.5 ± 3.9	0.3^c^

Abbreviations: ♀, Female; ♂, Male; GDS, Geriatric Depression Scale; HC, Healthy Control; ISC, Index of Sensitivity of Cueing; M, Mean; MDS‐UPDRS, The Movement Disorder Society – Unified Parkinson's Disease Rating Scale; MMSE, Mini Mental State Examination; SD, Standard Deviation.

^a^ One‐way ANOVA.

^b^ Chi‐square test.

^c^ Independent Sample *t* test.

^d^ Kruskal–Wallis test.

The criteria of Level 2 (comprehensive assessment) by Litvan et al. have been adopted for the purpose of evaluating participants with Parkinson's Disease under the diagnosis of Mild Cognitive Impairment. Within this context, each cognitive domain has been assessed with at least two neuropsychological tests. Patients who score one to two standard deviations below the normative values in two tests within one cognitive domain or in one test in two different cognitive domains are considered as having Parkinson's Disease Mild Cognitive Impairment (PDMCI).^3^ Hoehn Yhar Scale^19^ was used for staging the disease and Unified Parkinson's Disease Rating Scale (UPDRS)^20^ was used to determine the clinical level.

### 2.2 Classification of patients according to the type of memory disorder

Retrieval performance was examined based on the long‐term spontaneous recall and recognition scores of the Oktem Verbal Memory Processes Test.^21^ Based on this performance, individuals with PH‐MCI were classified into two groups: those with encoding failure (EF) and those with retrieval failure (RF). Here, the Index of Sensitivity of Cueing (ISC)^17^ was used as a criterion. According to this index, the sensitivity of individuals to cues and thus the level of retrieval disorders were determined. Patients with an ISC coefficient of 0.85 and above were included in the group with spontaneous RF. Patients who could not spontaneously retrieve and retrieve with cues multiple words, and had a ISC score of 0.85 and below, were included in the group with EF.

$$\begin{array}{c} \mathrm{ISC}=\left(\text{spontaneous}\;\text{recall}\;\text{score}-\text{total}\;\text{recall}\;\text{score}\right)/\\ \;\left(\text{spontaneous}\;\text{recall}\;\text{score}-\text{total}\;\text{item}\;\text{number}\right) \end{array}$$

### 2.3 Magnetic Resonance Imaging (MRI) procedure and analysis

**FIGURE 1:**
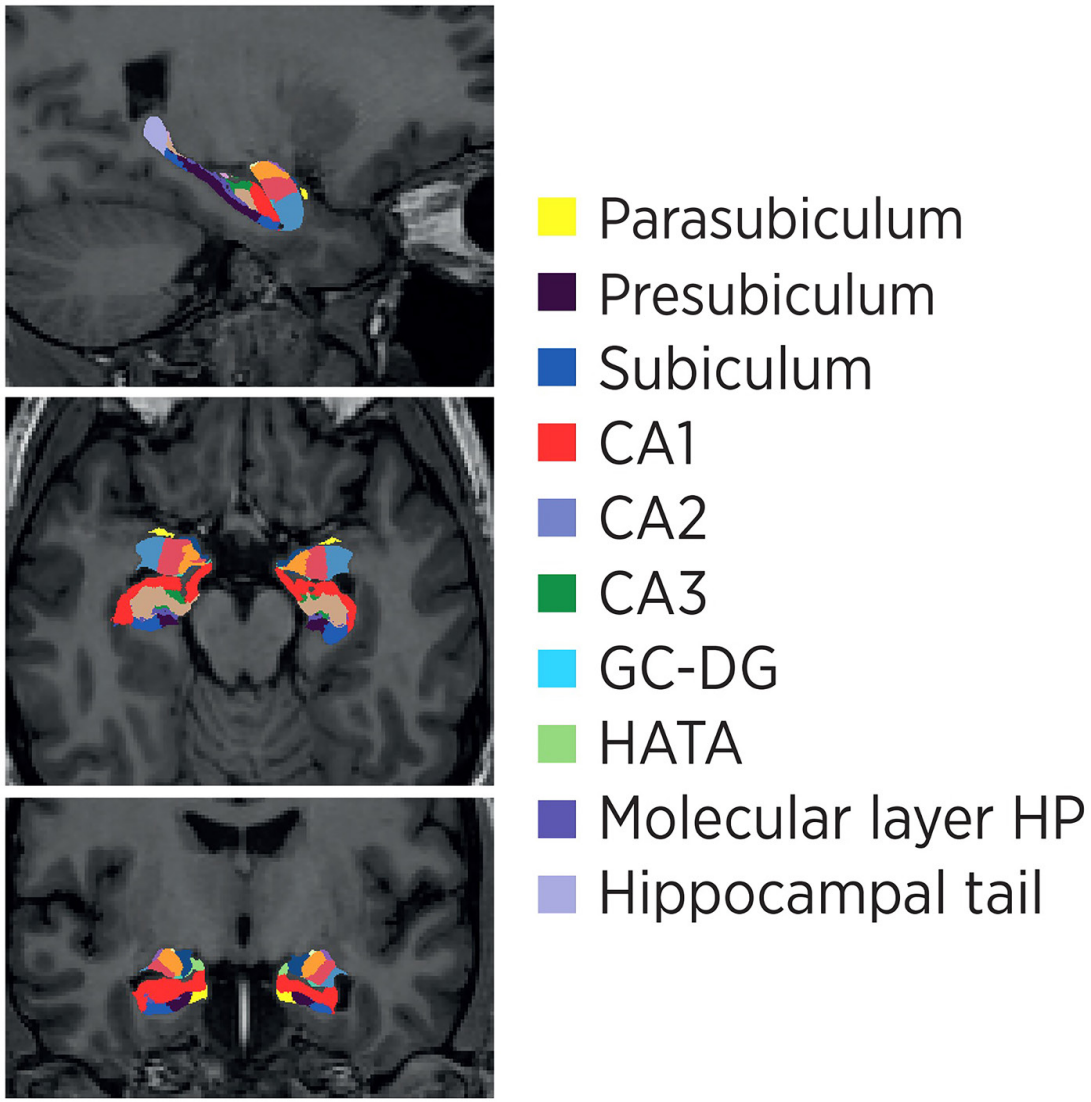
Subregions of hippocampal area.

Neuroimaging studies were performed using the Philips Ingenia CX 3 T Magnetic Resonance Imaging device, equipped with a 32‐channel head coil, at the, Istanbul Medipol University Hospital. The acquisition parameters for the anatomical T1‐weighted image were as follows: 190 slices with a repetition time/echo time (TR/TE) of 8.1/3.7, a field of view (FOV) dimension of 256 × 256 × 190 mm (FH × AP × RL), and a voxel size set at 1 × 1 × 1 mm. The preprocessing of participants' T1 images was carried out using an automatic reconstruction algorithm via the Freesurfer software (version 6.0.0). Subsequently, the new version of Freesurfer software (version 7.1.1) was used for segmentation of the subfields of the hippocampus. Volumes of various areas including subiculum, presubiculum, CA1, CA3, CA4, molecular layer, granule and molecular cell layer of the dentate gyrus (GC‐ML‐DG), whole hippocampus, as well as the head and body parts of the parasubiculum, fimbria error, and hippocampal tail were calculated using the segment_HA1 algorithm. In addition, “mean” values were formed by taking the average of the regions given as head and body. To standardize the volumes, each segmentation value was divided by the total intracranial volume (IVC) prior to statistical calculation (Figure 1).

Thickness calculation was continued via Freesurfer (version 6.0.0). After manually correcting the troubleshoots formed after the reconstruction in the cortex region (cortical editing), full width at half maximum (fwhm) images of the cortex were created using the qcache algorithm. Visualizations were then obtained through the qdec software.

### 2.4 Statistical analysis

Statistical analyses for comparing neuropsychometric test scores and volume data between groups, and for identifying differing brain regions, were performed using the IBM Statistical Package for the Social Science (SPSS) 21.0 software. To assess the adequacy of the sample size used in our study, a power analysis was conducted. This analysis was performed to determine the necessary sample size for *t*‐tests or ANOVA, using a power level of 0.80 and an alpha level of 0.05. As a result, the recommended total sample size was found to be at least 45, and accordingly, a total of 34 PD‐MCI patients and 16 healthy controls were selected. This study was designed to ensure that the chosen sample size has sufficient statistical power to detect the expected effect. The statistical analyses were conducted using parametric tests for groups demonstrating normal distribution and non‐parametric tests for groups not demonstrating normal distribution. The Shapiro–Wilk test was used to examine normality of the distribution. Demographic information of healthy control groups and patient groups were evaluated for consistent group distributions using one‐way ANOVA (Kruskal–Wallis test was conducted in case of abnormal distribution). Patient groups (two groups) were compared using independent group *t*‐tests (Mann–Whitney *U* Test in case of abnormal distribution) in terms of the degree of PD and general cognitive abilities.

Comparisons of mean neuropsychometric test scores for healthy and patient groups were made using One‐Way ANOVA (Kruskal–Wallis test in case of non‐normal distribution). Post‐hoc analyses (Tukey HSD, *p* < 0.05; Bonferroni corrected Mann–Whitney *U*, *p* < 0.017) were applied to identify between which two groups the difference exists in tests where a significant difference was found. Comparisons of mean MRI volumes of the patient groups (two groups) and the healthy control group were performed using One‐Way ANOVA (Kruskal–Wallis test in case of non‐normal distribution). Post‐hoc analyses (Tukey HSD; *p* < 0.05 due to the assumption of equality of variances) (Mann–Whitney *U* Test in case of non‐normal distribution) were applied in cases where a significant difference was found. In this study, *p* < 0.05 was considered statistically significant.

## 3 RESULTS

**FIGURE 2:**
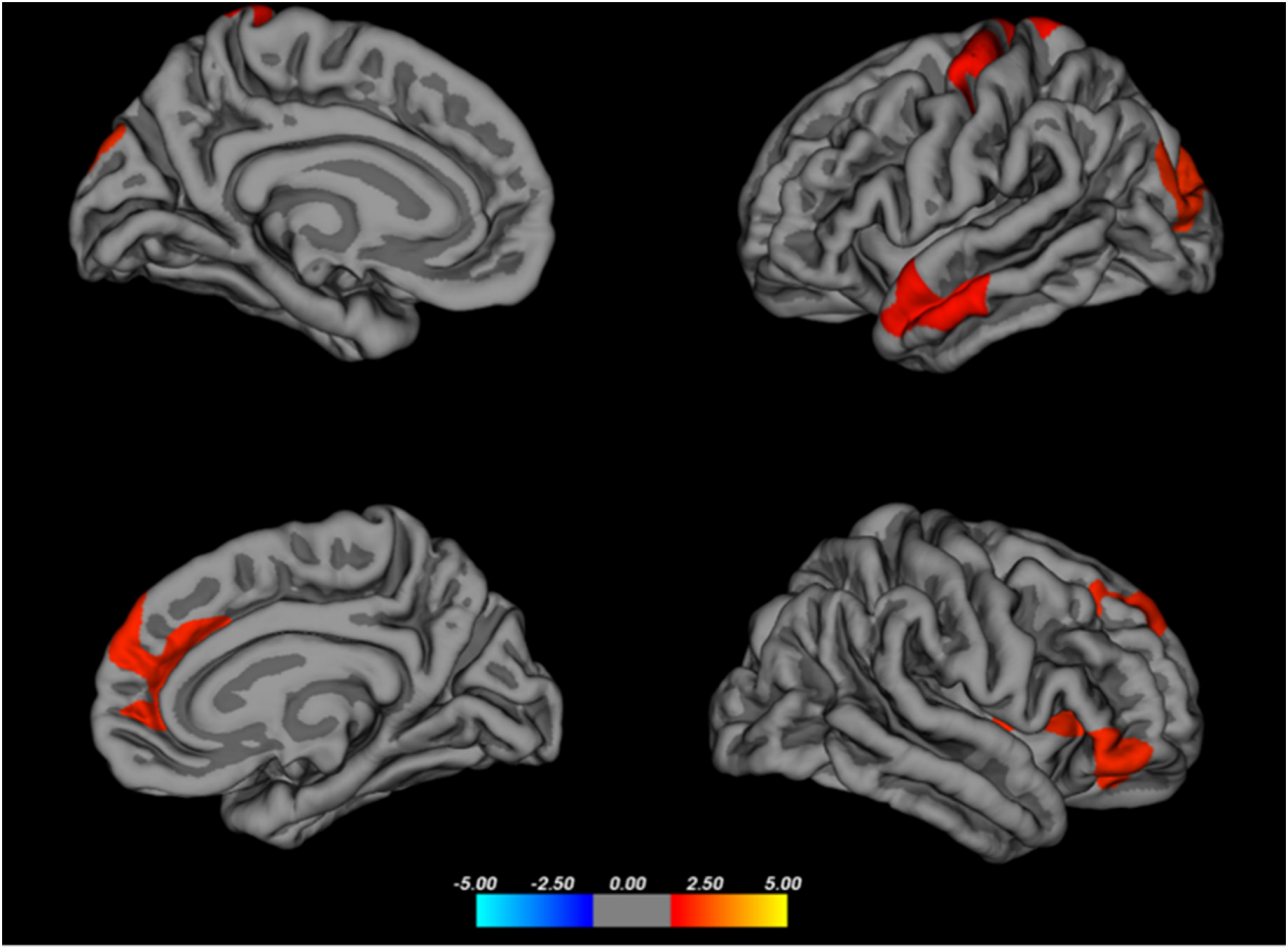
Thicker areas in the HC group compared to the RF group.

Differences in cortical thickness were observed in a wide area, which included the regions of the right hemisphere (rh) insula (14.28, 30.72, −27.10), rh lateral orbitofrontal (12.61, 57.13, −29.75), pars triangularis (18.19, 64.56, −22.09), rh medial orbitofrontal (−30.96, 91.47, −22.16), left hemisphere (lh) superior temporal (−29.99, 21.94, −51.13), lh postcentral (−1.08, −11.37, 63.57), lh inferior parietal (−6.16, −81.48, 17.20), and lh superior parietal (21.07, −95.01, 10.74) cortex regions (Table 2, Figure 2).

**TABLE 2:** The comparison of the cortical thickness between HC and RF groups.

Region	HC‐RF										
	Max	VtxMax	Size (mm^2^)	TalX	TalY	TalZ	CWP	CWPLow	CWPHi	NVtxs	WghtVtx
LH lateral occipital	4.396	87,146	1477.57	−28.8	−84.0	16.0	0.00870	0.00750	0.00990	2156	3708.46
LH middle temporal	1.980	40,053	1242.71	−61.4	−16.8	−16.0	0.02700	0.02490	0.02910	2002	2624.58
LH precentral	2.890	53,785	1229.30	−35.4	−15.1	60.8	0.02870	0.02660	0.03080	2905	4341.45
RH pars orbitalis	3.778	9143	1452.32	45.2	39.4	−11.3	0.01120	0.00990	0.01260	2850	4422.39
RH superior frontal	2.406	149,316	1432.28	9.2	36.6	26.3	0.01270	0.01130	0.01410	2568	3530.88

*Note*: lh hc is thicker than lh rf; *p* < 0.05; rh hc is thicker than rh rf; *p* < 0.05.

Abbreviations: HC, Healthy Control; LH, Left Hemisphere; RF, Retrieval Failure; RH, Right Hemisphere.

**FIGURE 3:**
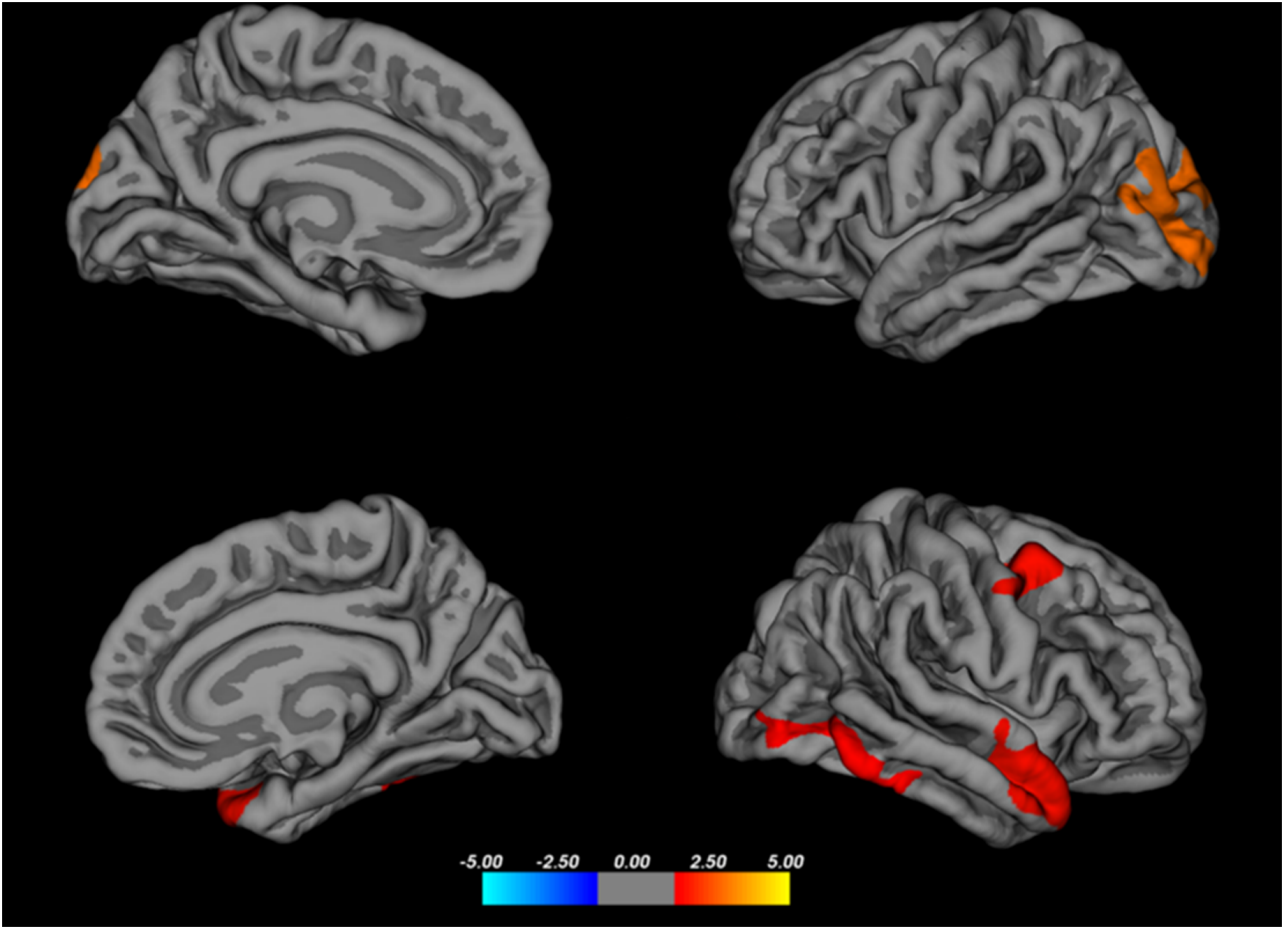
Thicker areas in the HC compared to the EF group.

Differences in cortical thickness were observed across a wide area, encompassing regions of the right hemisphere (rh) lateral occipital (45.97, −83.17, −7.34), rh middle temporal (59.45, 2.82, −28.89), rh temporal pole (34.23, 20.27, −37.50), rh precentral (56.56, 0.14, 43.69), left hemisphere (lh) inferior parietal (−42.29, −83.43, 29.29), and lh superior parietal (−13.96, −94.05, 23.70) cortex regions (Table 3, Figure 3).

**TABLE 3:** The comparison of the cortical thickness between HC and EF groups.

Region	HC‐EF										
	Max	VtxMax	Size (mm^2^)	TalX	TalY	TalZ	CWP	CWPLow	CWPHi	NVtxs	WghtVtx
LH lateral occipital	2.509	104,585	1919.73	−12.7	−91.7	22.3	0.00120	0.00080	0.00170	2780	4186.88
RH caudal middle frontal	2.769	82,716	1095.57	40.2	5.0	45.1	0.03070	0.02850	0.03290	1887	3046.90
RH superior temporal	3.598	18,716	1094.11	49.7	9.3	−18.9	0.03080	0.02860	0.03300	1870	3140.41
RH inferior temporal	2.777	97,739	1034.57	52.9	−57.3	−4.4	0.04340	0.04080	0.04600	1473	2103.07

*Note*: *p* < 0.05.lh hc is thicker than lh ef; *p* < 0.05; rh hc is thicker than rh ef.

Abbreviations: EF, Encoding Failure; HC, Healthy Control; LH, Left Hemisphere; RH, Right Hemisphere.

When comparing the hippocampal subfields between the groups, the HC group exhibited a larger volumetric size in the following regions compared to the EF group: Left CA1 body, Left CA3 body, Left central nucleus, Left medial nucleus, Right cortical nucleus, Left CA3 mean, Left CA4 body, Left CA4 mean, Left molecular layer body, Left GCMLDG body, and Left whole hippocampal body. Similarly, in the comparison between the RF and EF groups, the regions: Left CA1 body, Left CA3 body, Left medial nucleus, Left CA3 mean, Left CA4 body, and Left whole hippocampal body were statistically larger in favor of the RF group. No significant difference was observed in the hippocampal subfields between the HC and RF groups (Table 4).

**TABLE 4:** Comparison of the ICV normalized hippocampal subfields between the groups.

Subfield	HC (M ± SD)	RF (M ± SD)	EF (M ± SD)	HC‐EF (*p*)	RF‐EF (*p*)
**Left CA1 body**	0.000087 *± 0.000017*	0.000086 *± 0.000020*	0.000068 *± 0.000017*	0.014^a^	0.014^a^
**Left CA3 body**	0.000062 *± 0.000011*	0.000062 *± 0.000013*	0.000050 *± 0.000011*	0.024^a^	0.017^a^
Left central nucleus	0.000030 *± 0.000006*	0.000029 *± 0.000008*	0.000024 *± 0.000005*	0.048^a^	non‐sig.^a^
**Left medial nucleus**	0.000014 *± 0.000004*	0.000013 *± 0.000005*	0.000010 *± 0.000002*	0.022^a^	0.033^a^
Right cortical nucleus	0.000018 *± 0.000004*	0.000017 *± 0.000005*	0.000015 *± 0.000004*	0.035^a^	non‐sig.^a^
**Left CA3 mean**	0.000071 *± 0.000011*	0.000072 *± 0.000013*	0.000061 *± 0.000012*	0.024^b^	0.049^b^
**Left CA4 body**	0.000083 *± 0.000014*	0.000083 *± 0.000016*	0.000070 *± 0.000016*	0.014^b^	0.025^b^
Left CA4 mean	0.000085 *± 0.000014*	0.000085 *± 0.000017*	0.000074 *± 0.000016*	0.016^b^	non‐sig.^a^
Left molecular layer body	0.000155 *± 0.000029*	0.000152 *± 0.000032*	0.000129 *± 0.000027*	0.009^b^	non‐sig.^a^
Left GCMLDG body	0.000093 *± 0.000017*	0.000092 *± 0.000019*	0.000079 *± 0.000020*	0.019^b^	non‐sig.^a^
**Left whole hippocampal body**	0.000818 *± 0.000145*	0.000799 *± 0.000160*	0.000699 *± 0.000139*	0.014^b^	0.042^b^

*Note:* The comparison of the HC‐RF values was not included in the table due to the absence of statistically significant results.

Abbreviations: EF, Encoding Failure; HC, Healthy Control; M, Mean; RF, Retrieval Failure; SD, Standard Deviation.

^a^ Tukey HSD.

^b^ Mann–Whitney Test.

## 4 DISCUSSION

Significant differences were found in cortical thickness and subcortical volumes when comparing the HC, EF, and RF groups across various regions. A key observation was the distinct atrophic patterns differentiating the EF and RF groups. Specifically, compared to the HC group, the EF group demonstrated differentiation in cortical thickness in the temporo‐parieto‐occipital region, whereas this manifestation was different for the RF group. The RF group, when compared to the HC group, exhibited cortical thinning in the frontotemporal regions. However, a common denominator for both the EF and RF groups is the thinning observed in the lateral occipital areas. In the hippocampal volume comparisons, the EF group was found to have a significant volume reduction compared to both the HC and RF groups. A notable difference was particularly evident in the subfields of the hippocampus. This observation was consistent with the study's expectations and objectives. This study identifies distinct cortical atrophy patterns in individuals with encoding and retrieval failures, highlighting the temporo‐parieto‐occipital thinning in encoding failure and frontotemporal thinning in RF, with both groups showing thinning in lateral occipital areas. Particularly, a significant hippocampal volume reduction in the EF group underscores the differential impact of cognitive dysfunctions on brain structure, aligning with the study's objectives to elucidate specific neuroanatomical correlates of memory processing failures.

### 4.1 Relationship between hippocampal atrophy and memory impairment in PD‐MCI

The widely acknowledged consensus is that the hippocampus has a significant role in episodic memory. As such, elucidating the association between hippocampal dysfunction and episodic memory disorders in PD is crucial. This understanding is fundamental for identifying the origins and etiology the progression of memory impairments within the PD context.^22^, ^23^

The hippocampal formation consists of the Cornu Ammonis (CA1, CA2, CA3, CA4) regions, dentate gyrus, and subiculum.^24^ Numerous intriguing studies, utilizing high‐resolution functional MRI with healthy participants, have demonstrated that entry regions of the hippocampus such as CA2, CA3, and the dentate gyrus are implicated in the encoding and learning of information. Conversely, subregions like the subiculum, CA1, and CA3 are involved in the retrieval of newly learned information.^23^, ^24^, ^25^, ^26^, ^27^, ^28^ Two recent studies aimed to reveal the relationship between changes in hippocampal volume and memory performance in Parkinson's patients. The findings obtained in this direction demonstrate a volumetric decrease in the CA2, CA3, CA4, and dentate gyrus regions in the Parkinson's group compared to healthy controls. This situation has been associated with the decrease in learning scores.^24^, ^29^

In another study investigating hippocampal subfields in Parkinson's patients, it was found that the radial distances of CA1, CA3, and the subiculum were correlated with both short‐term and long‐term spontaneous recall abilities. Meanwhile, the thickness of the subiculum was observed to be associated with recognition score performance.^23^, ^24^ In a longitudinal study conducted with PD‐MCI patients, it was observed that patients' bilateral hippocampal volumes significantly diminished over an 18‐month period, potentially correlating with cognitive decline. However, because the volumetric decrease witnessed in this study was not exclusive to the hippocampus, it was not feasible to establish a direct association between this decrease and episodic memory impairment in individuals with PD‐MCI.^30^ In another recent neuroimaging study, it was shown that in early PD, frontostriatal areas, entorhinal cortex, hippocampus, and surrounding medial temporal areas show morphological changes, and these areas are important in cognitive impairment. However, the results shown by Pirogovsky‐Turk et al. indicate that the volume loss in frontostriatal areas in PH‐MCI is associated with executive functions.^31^ They have stated that poor learning and encoding of information is not only due to executive dysfunction but also associated with the volumetric decrease of medial temporal areas, especially the hippocampus.^31^ Similarly, Han et al. distinguished a group of patients with MCI into groups with EF and RF. Comparing the gray matter volumes of these two groups, researchers have found a significant volumetric difference in the bilateral hippocampus regions of the group with encoding failure compared to the normal control group. They state that the encoding failure group is similar in nature to brain atrophy observed in Alzheimer's disease.^32^ Studies generally agree on hippocampal atrophy in Parkinson's patients.^24^, ^29^, ^33^, ^34^ However, the level of atrophy is not as significant as in Alzheimer's patients.^35^, ^36^

Our findings, in line with the literature, demonstrate a volumetric decrease in the subregions of the hippocampus, namely CA1, CA3, and CA4 areas, in the EF group compared to the HC and RF groups. In contrast, we were unable to find any volumetric differences in subcortical regions between the HC and RF groups. In accordance with the literature, our findings show a volumetric deficiency in the sub‐areas of the hippocampus, namely CA1, CA3, and CA4 regions, in PD‐MCI patients who have difficulty in learning and encoding information. This aligns with previous reports from Beyer et al,^23^ Györfi et al,^24^ and Pereira et al.^29^ Based on our findings, we believe that PD‐MCI patients who have difficulty in recording and encoding demonstrate a volumetric decrease in the sub‐areas of the hippocampus, similar to brain atrophy observed in Alzheimer's type dementia.

### 4.2 The pattern of cortical atrophy observed in PD‐MCI with EF patients

In the PD‐MCI with EF group, when compared with HC, we found significant atrophy in the cortical thickness of the left lateral occipital; right caudal middle frontal, superior temporal, and inferior temporal. Episodic memory disorders in PD‐MCI are generally associated with RF.^17^ This is evident as PD‐MCI patients score lower on memory tests that require spontaneous recall compared to healthy individuals. However, they are able to significantly improve their performance on tasks that involve recognizing previously learned information.^37^, ^38^ This is generally interpreted as seconder deficiency to attention and executive function deficiencies due to the disturbance in dopaminergic activation in the frontostriatal circuit.^17^ Other evidence has shown that the origin of memory disorder in PD‐MCI from either posterior cortical or frontostriatal can be very valuable in terms of prognosis and pathophysiology.^38^, ^39^ Especially in recent years, structural MRI studies in PD‐MCI show a correlation between hippocampal atrophy and memory disorders (18, 36). Therefore, while hippocampal dysfunction plays a significant role in memory defects in PD‐MCI, it also contributes to memory deficiencies in other cortical regions.^40^ In recent years, there has been an increase in studies that demonstrate thinning in cortical thickness or decrease in functional connectivity in PD‐MCI and relate this to memory deficiencies. In particular, the effects of structural and functional connectivity of other brain networks, with frontoparietal networks as the focus point, on memory processes are revealed.^16^, ^40^, ^41^, ^42^, ^43^

An fMRI study illustrating the functional interconnections within parietal regions indicated that the interplay between the dorsolateral and ventrolateral prefrontal regions, in conjunction with posterior brain regions – including the lateral parietal lobe and medial temporal lobe – bolster the retrieval and recognition of relationally encoded information during episodic memory processes.^44^, ^45^, ^46^, ^47^ Ray and colleagues, examining this situation on the frontoparietal network, showed that frontal and parietal regions support cognitive control and interact with the parietal memory network, supporting encoding and retrieval abilities of episodic memory processes.^44^ Segura and colleagues demonstrated a pattern of posterior atrophy characterized by cortical thinning in bilateral superior parietal, supramarginal, inferior temporal, parahippocampal gyrus, fusiform gyrus, and precuneus in PD‐MCI patients.^42^ They summarized this situation as an important finding in terms of cognitive disorders and progression to dementia in PD‐MCI, with a pattern of posterior atrophy in PD‐MCI patients that extends to frontal regions but mostly involves posterior parietal–temporal areas. Another research observed a progressive decrease in connectivity over the frontoparietal network, which includes regions involved in recognition memory in Parkinson's patients. However, this study revealed that the connection between frontal areas is preserved.^42^

Research delineating the interaction between regions identified within the Default Mode Network (DMN) and the memory circuit disclosed a diminished functional connection between the posterior cingulate cortex region embedded within the DMN and the left medial temporal lobe. This diminished connection was associated with an adverse influence on verbal memory.^40^ The findings obtained from different studies point to the exhibition of hypoperfusion in the parietal‐occipital network in PD‐MCI.^48^, ^49^ This indicates that the dysfunction of the posterior PMN underlies memory deficiencies in PD‐MCI.^50^ Our findings, in line with the literature, provided evidence that cortical differentiation in PD‐MCI is decisive in distinguishing the form of memory disorder. In this context, there is a cortical thinning towards especially the posterior areas in the EF‐PD‐MCI group. We think that this situation makes it difficult to learn new information and this clinically manifests as a deficiency in recognition memory. This situation is an important clue for the transformation to dementia.^50^, ^51^

### 4.3 In patients with RF, the pattern of cortical atrophy observed in those with PD‐MCI

Significant atrophy was found in the lh lateraloccipital, lh middle temporal, lh precentral; rh parsorbitalis, rh superiorfrontal regions when the RF PD‐MCI group was compared with HC. According to the RF hypothesis, the typical PD memory profile arises from attention and executive function disorders. This is explained by pathophysiological changes in fronto‐striatal networks associated with memory retrieval.^1^, ^52^, ^53^ However, recognition memory is relatively preserved in such memory disorders.^1^ In their research, Bezdek et al. reported that decreased functional connectivity between the precuneus and the superior parietal cortex was important in explaining the RF based on attention disorder in PD‐MCI patients.^1^ In another study where MCI patients with RF were examined, Han and colleagues found decreases in gray matter volumes in the left thalamus, right superior frontal lobe, right superior temporal lobe and right middle cingulum. However, the group with RF exhibits similar characteristics with the healthy control group in terms of hippocampal volume.^32^ This is consistent with our findings. Our RF group exhibits a deficiency in spontaneous memory retrieval due to the deficiency in executive functions. This matches with the regions showing cortical thinning being close to the frontal areas and hippocampal volumes being at a similar level with healthy controls.

However, we identified a common convergence in both our PD‐MCI groups. The lateral occipital cortex area showed similar levels of cortical thinning in both EF and RF areas. This finding supports a new network approach to memory functions in the literature: Inferior parietal lobule (IPL) and temporoparietal junction (TPJ).^54^ The term TPJ has been used for activations observed largely in the IPL and dorsal areas of the posterior superior temporal lobe. However, it is also partially extends to the mid temporal gyrus and lateral occipital lobe.^54^ It has been shown that the IPL/TPJ is generally activated during the retrieval of autobiographical or other episodic memories.^10^, ^55^ In particular, it has been shown that the posterior angular gyrus area, which is structurally connected to the medial temporal lobe memory system, is associated with IPL/TPJ activity.^54^ The relationship between IPL/TPJ network activation and attention and memory is still under discussion, but different regions of the network are thought to be responsible for attention and episodic memory.^54^ The effectiveness of the angular gyrus and lateral occipital cortex regions in memory processes has intrigued researchers who explain learning over schematic representation. Recent research reported that the neurobiological bases underlying learning processes related to the schema are particularly on the connections between ventromedial prefrontal cortex, hippocampus, angular gyrus and lateral occipital cortex brain regions.^47^, ^56^, ^57^ Our findings support the effectiveness of the lateral occipital cortex in memory processes in the context of contributing to the literature, but we need more comprehensive research on how much it is involved in the task.

The findings of this study suggest that the patterns of cortical and hippocampal atrophy may serve as significant determinants in the diagnosis of PD‐MCI patients. Clinically, these findings could be particularly useful in the early stages of the diagnostic process. For instance, observing specific atrophies may aid in monitoring and tracking the cognitive status of patients. Furthermore, detecting these changes could facilitate the development of personalized treatment strategies, thereby enabling the implementation of more targeted therapeutic approaches.

## 5 CONCLUSION

Our findings showed volumetric loss in the subfields of the hippocampus only in the EF group. In addition, we showed that the thinning in cortical thickness affected different areas between the RF and EF groups. Cortical thinning was especially prominent in the frontal structures in the RF group. However, the presence of cortical thinning in the temporal and occipital regions in the EF group indicates that there is not a uniform pattern in terms of memory destruction in PD‐MCI, that there may be possible subgroups in terms of cognitive destruction of the disease and that these may be associated with cortical/subcortical destruction in different patterns. The subgroups are likely to have different trajectories in terms of progression to dementia, and it may be beneficial to predict this course with a simple neuropsychometric assessment as we described.

Another finding of our study is that in addition to different cortical atrophy patterns accompanying different memory impairment patterns (encoding disorder and retrieval disorder), there are also brain areas that show common destruction (lateral occipital cortex). When all our findings are evaluated together, we think that it will contribute to the better understanding of the neurobiological origins of the memory problems observed in PD‐MCI and the development of our knowledge about how the memory network works.

## FUNDING INFORMATION

No funding was received to support this research.

## CONFLICT OF INTEREST STATEMENT

The authors declare no potential conflicts of interest.

## Data Availability

The data supporting this study are available from the corresponding author upon reasonable request.
